# Conditionally Reprogrammed Cells Preserve Cellular Diversity and Permit Genetic Manipulation: Implications for Cancer Heterogeneity and Tissue Regeneration

**DOI:** 10.17161/sjm.v3i1.24566

**Published:** 2026-02-04

**Authors:** Shuang Fang, Guangzhao Li, Dilber Nurmurmet, Xiaokui Mo, Chen Fei, Sujata Choudhury, Nancy Palechor-Ceron, Richard Schlegel, Xuefeng Liu, Jenny Li

**Affiliations:** 1 Department of Pathology, Georgetown University Medical Center, Washington, D.C. 20057, USA;; 2 Comprehensive Cancer Center, Wexner Medical Center, The Ohio State University, Columbus, Ohio 43210, USA;; 3 Department of Biostatistics and Bioinformatics, Comprehensive Cancer Center, Wexner Medical Center, The Ohio State University, Columbus, Ohio 43210, USA;; 4 Departments of Pathology, Comprehensive Cancer Center, Wexner Medical Center, The Ohio State University, Columbus, Ohio 43210, USA

**Keywords:** conditionally reprogrammed cells (CRC), cancer heterogeneity, tissue repair, patient-derived cell models

## Abstract

Our previous study demonstrated that a Rho kinase inhibitor (Y-27632), in combination with irradiated fibroblast feeder cells, enables both normal and tumor epithelial cells from various tissues to proliferate indefinitely in vitro, without the need for exogenous viral or cellular gene transduction. These cells are referred to as Conditionally Reprogrammed Cells (CRCs). This approach has shown great promise for applications in regenerative and personalized medicine. In the present study, we first investigated whether CRCs could be genetically manipulated and stably express exogenous genes. Using lentiviral transduction of GFP, we successfully infected human foreskin keratinocytes (HFKs) cultured under CRC conditions. GFP expression was stably maintained over multiple passages and through freeze–thaw cycles. Similar stable GFP expression was also observed in CRCs derived from human tumor specimens, including neuroendocrine cervical carcinoma and prostate cancer. We next explored the use of GFP-labeled CRC-HFKs in co-culture systems with tumor-derived CRCs to evaluate colony formation and cellular heterogeneity. Our data demonstrated that CRCs from different individuals can form heterogeneous colonies, supporting the potential of CRC-based systems for modeling tissue development, regeneration, and tumor heterogeneity. To optimize the CRC platform for translational applications, we further assessed human fibroblasts as an alternative to the mouse feeder layer, using GFP-expressing CRCs as a functional readout. Finally, we demonstrated the capacity for gene knockdown in CRCs by successfully silencing p53 and HPV16 E6 in cervical cancer-derived CRCs using shRNA lentiviral vectors. These results collectively establish that the CRC method supports stable genetic manipulation and underscore its potential for mechanistic studies, disease modeling, and development of cell-based therapeutic strategies.

## Introduction

Traditionally, cancer research and drug development utilized cancer cell lines [1–5] and genetically engineered mouse models (GEMM) [6]. In recognition of the many limitations of these cancer models, patient-derived models of cancer (PDMCs) were developed recently, including patient-derived xenografts (PDXs), CRCs, and organoids. In recent years, PDX models faithfully recapitulate the original patient genetic profile, gene expression patterns, and tissue histology [7, 8], and are widely recognized as a more physiologically relevant preclinical model. Despite their benefits, PDX models are limited by their inherent variability, low throughput, and lack of growth in vitro. Development of stable PDX lines remains a challenge due to murine stromal outgrowth, lineage commitment, and limited differentiation potential. Overall, it takes 2–5 months for PDX expansion at a relatively high cost for mice and their care. The laboratories of Clevers and others successfully established organoid cultures initially from mouse tissues, and subsequently from human specimens [9–14]. Organoid cultures provide a platform to investigate basic biology for cancers, to identify drug targets, and to study drug resistance [15–17]. Organoid cultures are established using 3-dimensional growth of epithelial cells in Matrigel^™^, providing an opportunity for their application to personalized and regenerative medicine. Challenges include a requirement for precise tumor tissue sampling for organoid cultures, as this culture system propagates both normal and cancer cells. Overall, it takes 4–6 weeks to provide enough cells for low-throughput drug screening. It remains a challenge to establish a single model system that is rapid and simple to perform with a high rate of success from various clinical samples (surgical specimens, needle biopsies, cryopreserved tissues, blood, and urine).

Our previous study demonstrated that a Rho-associated kinase inhibitor (Y-27632), in combination with irradiated fibroblast feeder cells, enables both normal and tumor epithelial cells from a variety of tissues to proliferate indefinitely in vitro, without the need for exogenous viral or cellular gene transduction [18–27]. These cells, termed Conditionally Reprogrammed Cells (CRCs), undergo rapid and reversible reprogramming of the entire cell population [18–21, 27]. These CRCs can be used for establishing xenografts [26, 27], patient-derived xenograft cell lines [28], cell cultures from PDXs, and organoid cultures. The CRC method has shown great promise for regenerative and personalized medicine, cancer biology [19–22, 27, 29–40].

To advance human therapeutic applications, the development of an animal-free CRC culture system is highly desirable to eliminate exposure to mouse cells and animal-derived serum. A system that is both feeder-free and genetically tractable would significantly enhance the translational potential of CRCs for cell-based therapies and tissue engineering. It is therefore crucial to investigate whether CRCs can be genetically manipulated and whether human-derived components can effectively substitute for the mouse-based system.

Previous studies have shown that human ESCs can be successfully maintained on human embryonic fibroblasts or adult fallopian tube epithelial cells [41]. Here, we evaluated whether human foreskin fibroblasts (HFFs) could replace mouse feeders in the CRC system by co-culturing GFP-labeled human foreskin keratinocytes (HFKs) with HFFs and J2 cells under CRC conditions.

Cancer cell heterogeneity refers to the existence of diverse subpopulations of cancer cells within the same tumor or among tumors from different patients. This heterogeneity arises from genetic mutations, epigenetic alterations, and interactions with the tumor microenvironment, leading to variations in cell morphology, gene expression, proliferation rates, and therapeutic responses. Such complexity poses significant challenges for cancer diagnosis, treatment, and drug resistance. Since CRC allows primary cancer cells to propagate from patient-cancer tissues, it would be critical to evaluate whether CRCs maintain cell diversity using mixed cell populations with either unique markers or labeled cells.

Taken together, these findings provide a strong rationale for further development of humanized, genetically tractable CRC systems for use in regenerative medicine, disease modeling, and cell-based therapeutic strategies. The ability of CRCs to preserve cellular diversity indicates a promising utility of this technology in cancer cell heterogeneity and translational oncology.

## Materials and Methods

### Cell Culture

GUMC-395 was established from a 26-year-old woman diagnosed with large-cell neuroendocrine carcinoma of the cervix at Georgetown Center for Cell Reprogramming and described in the previous publication [42]. The tumor exhibited a Ki-67 index of 93% and was HPV16-positive. Molecular analysis revealed a p53 copy loss and two distinct mutations (R273C and C141R), as well as 100-fold amplification of the Myc gene. GUMC-030 was derived from a radical prostatectomy specimen of a 57-year-old patient with stage T3b, Gleason 7 adenocarcinoma at Georgetown Center for Cell Reprogramming and described in the previous publication [43]. GUMC-395 cells, derived from neuroendocrine cervical carcinoma, were maintained in collagen-coated flasks in F+Y medium [26, 44]. The F+Y medium consists of F medium [3:1 (v/v) F-12 Nutrient Mixture (Ham) and Dulbecco’s Modified Eagle Medium (Invitrogen)] supplemented with 5% fetal bovine serum (FBS), 0.4 μg/ml hydrocortisone (Sigma-Aldrich), 5 μg/ml insulin (Sigma-Aldrich), 8.4 ng/ml cholera toxin (Sigma-Aldrich), 10 ng/ml epidermal growth factor (Invitrogen), and 24 μg/ml adenine (Sigma-Aldrich), with the addition of 5–10 μM Y-27632 (Enzo Life Sciences). Human foreskin keratinocytes (HFKs) were cultured from neonatal foreskin samples as previously described and maintained in two types of media: (1) keratinocyte growth medium (KGM) supplemented with gentamycin (50 μg/ml), and (2) conditional reprogramming culture (CRC) medium using a fibroblast feeder system, co-cultured with irradiated (3000 rad) Swiss 3T3 fibroblasts (J2 strain) in F+Y medium. All cells were maintained at 37 °C in a humidified incubator with 5% CO_2_.

### Feeder Cells

Swiss 3T3-J2 mouse fibroblasts [26, 44] were cultured in complete DMEM (Dulbecco’s Modified Eagle Medium, Life Technologies, Carlsbad, CA) supplemented with 10% FBS, 100 μg/ml penicillin, 100 μg/ml streptomycin, and 100 μg/ml glutamine. Following irradiation (3000 rad), the J2 feeder cells were seeded at approximately 70% confluency into the desired flasks or dishes and used the following day. All cells were maintained at 37 °C in a humidified incubator with 5% CO_2_.

### Separation of Feeder and Epithelial Cells

Differential trypsinization was performed to remove feeder fibroblasts from epithelial cells. Briefly, cells were rinsed with DPBS (Invitrogen) and incubated at room temperature for 30 seconds with 0.05% Trypsin-EDTA (Invitrogen). The flask or dish was gently rocked and monitored under a microscope to ensure maximum removal of fibroblasts. The remaining cells were washed with DPBS. Epithelial cells were then trypsinized for 3–5 minutes at 37 °C. The culture vessel was gently tapped, and the enzymatic reaction was stopped by adding DPBS containing 10% FBS. The cell suspension was centrifuged, and the pellet was resuspended in F medium.

### Transduction of HFK, GUMC-030, and GUMC-395 Cells

HFK, GUMC-395, and GUMC-030 cells were transduced with lentiviral GFP constructs to establish stable cell lines. GUMC-395 cells were additionally transduced with lentiviral shRNA constructs targeting TP53 and HPV E6 (Santa Cruz Biotechnology, Inc.), and stable lines were established through serial passaging. The protocol was as follows: Day 1: Cells were seeded into 12-well plates in 1 mL of complete medium and incubated overnight to reach ~50% confluence; Day 2: Complete medium containing 5 μg/mL Polybrene^®^ (sc-134220) was added to each well. Lentiviral particles were thawed, gently mixed, and added to the wells. Plates were gently swirled and incubated overnight; Day 3: Medium was replaced with fresh complete medium (without Polybrene), and cells were incubated overnight. Day 4: Cells were split 1:3 to 1:5, depending on cell type, and incubated in complete medium for an additional 24–48 hours; Days 5–6 and onward: Puromycin dihydrochloride (sc-108071) was added for selection of stable shRNA-expressing clones.

### Cell Counting

Cells were counted using the Invitrogen Countess^™^ Automated Cell Counter. After centrifugation and resuspension in medium, 10 μl of the cell suspension was mixed with 10 μl of 0.4% Trypan Blue stain (Life Technologies) in a 0.5 ml microcentrifuge tube. A 10 μl aliquot of the mixture was loaded onto the counting slide and analyzed using the automated counter.

### RT-PCR

Total RNA was extracted using the RNeasy Kit (Qiagen, Valencia, CA; Cat. No. 74106). A total of 2.5 μg of RNA was used for cDNA synthesis and stored at −20 °C. Quantitative RT-PCR (qRT-PCR) was performed using the iCycler MyiQ system (Bio-Rad, Hercules, CA, USA). The following primers were used: HPV E6: Forward: 5′-ACAGAGCTGCAAACAACTAT-3′

Reverse: 5′-TTGCAGTACACACATTCTAA-3′; TP53: Forward: 5′-GGAGCCGCAGTCAGA TCCTA-3′ Reverse: 5′-GGGGACAGAACGTTGTTTTC-3′; GAPDH (reference): Forward: 5′-TCTCCTCTGACTTCAACAGC-3′ Reverse: 5′-GAAATGAGCTTGACAAAGTG-3′. qRT-PCR was performed using iQ SYBR Green Supermix (Bio-Rad). Data were normalized to GAPDH using the ΔΔCT method according to the manufacturer’s instructions.

### Pathway analysis

Transcriptome was performed in our previous studies [20, 45]. DEG (differentially expressed genes) from C1/C2 and C3/C6/C8 subgroups were analyzed with the QIAGEN IPA program. The heatmap was constructed using MultiExperiment Viewer (MeV) v4.9.0. Both genes and samples were grouped via hierarchical clustering analysis (HCL) using Euclidean distance metrics.

## Results

### CRC Conditions Promote Proliferation of Normal and Tumor Cells

**Figure 1.**
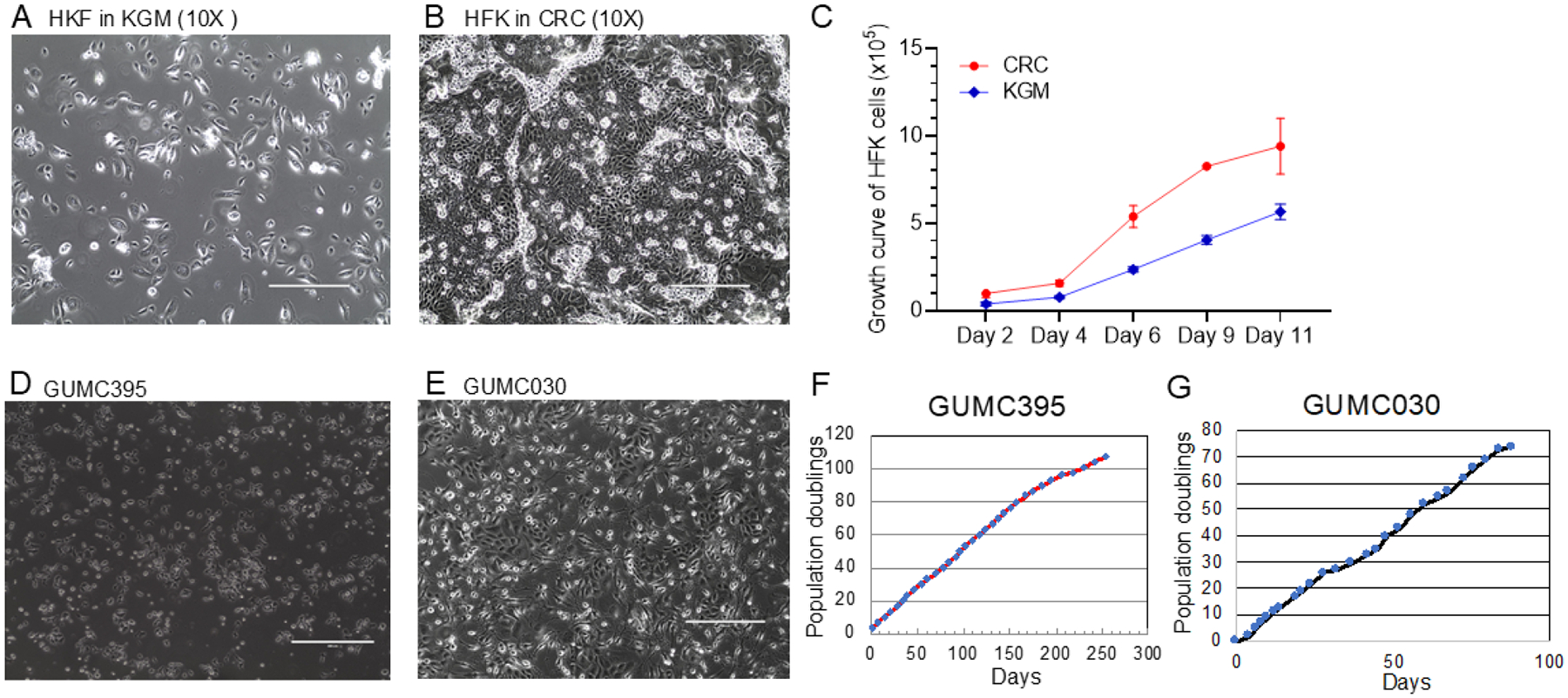
CRC conditions promote the proliferation of both normal and tumor cells. (A) Cell morphology of HFKs in KGM condition (Day 4). (B) Cell morphology of HFKs cultured in CRC condition (Day 4). (C) Growth curves of HFKs under CRC and KGM condition. (D) Cell morphology of GUMC-395 (cervical cancer) in CRC condition. (E) Cell morphology of GUMC-030 (prostate cancer) cells in CRC condition. (F) Growth curve of GUMC-395 cells in CRC condition. (G) Growth curve of GUMC-030.

We first evaluated the growth advantage of human foreskin keratinocytes (HFKs) under CRC conditions compared to synthetic medium (KGM). As shown in Figure 1A & 1B, HFKs displayed distinct morphologies on Day 4 in KGM versus CRC, suggesting enhanced proliferation in CRC medium. To quantify this, we plated 5,000 cells per well in 6-well plates and counted cell numbers every 2–3 days. Growth curves (Figure 1C) confirmed that CRC conditions significantly promoted proliferation. Additionally, we assessed tumor-derived cells under CRC conditions. Figure 1D & 1E show representative morphologies of neuroendocrine cervical cancer cells (GUMC-395) and primary prostate cancer cells (GUMC-030), both of which grew well in CRC conditions, respectively. Figure 1F & 1G show the growth curves of these two tumor cell lines that were established using the CRC culture technique as previously described.

### Stable Genetic Manipulation of Normal CRCs with GFP

**Figure 2.**
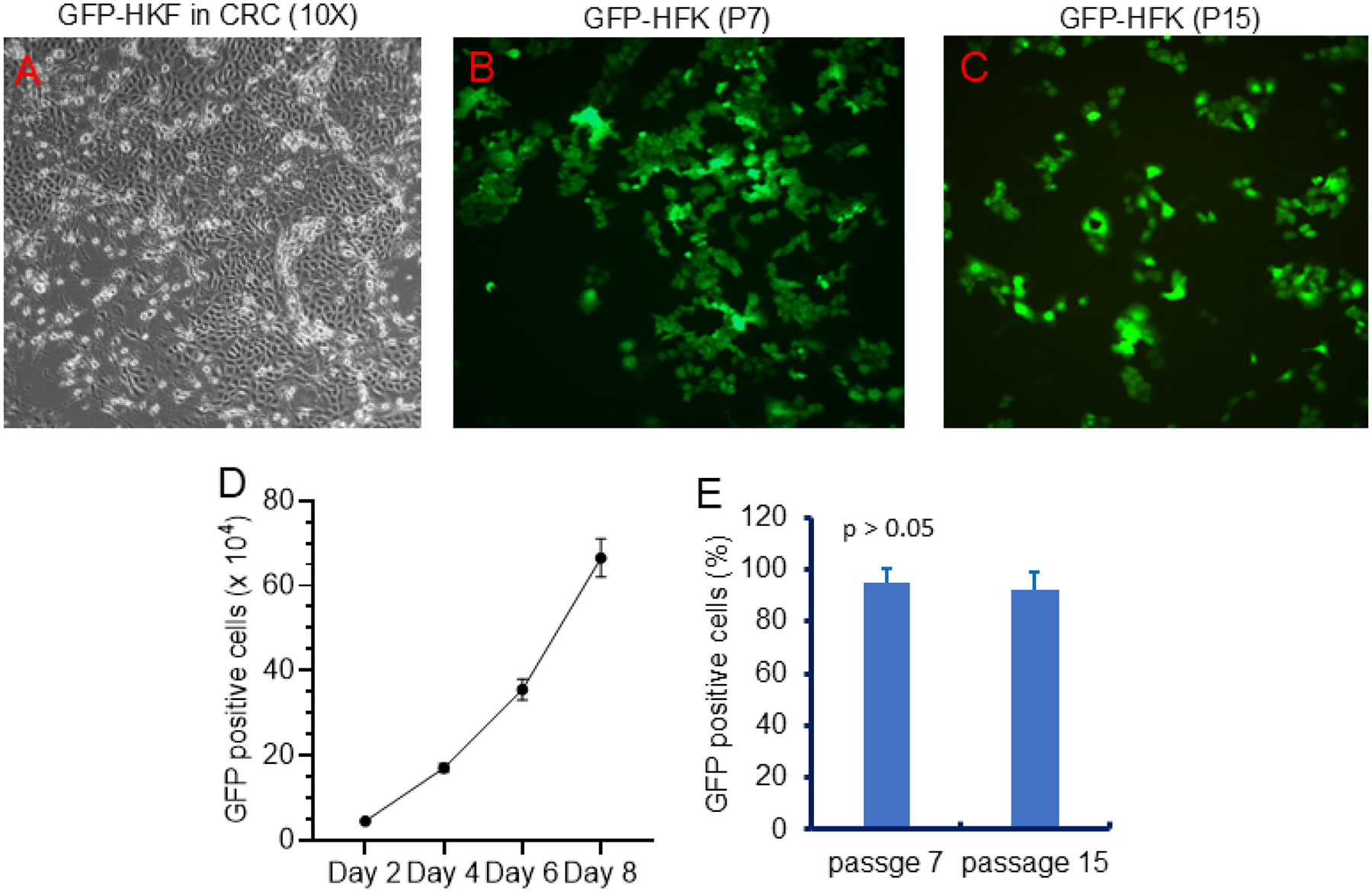
Genetic manipulation of normal CRCs with GFP. Stable GFP-expressing HFKs were established using lentiviral infection. GFP was maintained after passaging and freeze-thaw processing. (A) Phase contrast microscopy of GFP-HFKs. (B) IF microscopy of GFP-HFK at early passage (p7). (C) Fluorescent microscopy of passed GFP-HFK (p15). (D) Cell proliferation curve of GFP-HFK. (E) GFP positivity during passages of GFP-HFKs under CRC condition.

To test whether normal CRCs can be genetically modified, we infected HFKs cultured under CRC conditions with lentiviral GFP particles. As shown in Figure 2, the cells expressed high levels of GFP, which were maintained after more than five passages (1:4 or 1:8 split ratio) and after freeze-thaw cycles. This confirms that GFP-labeled HFK-CRCs can be stably maintained and used for downstream applications.

### Mixed Cultures of GFP-HFKs and Prostate Cancer CRCs

**Figure 3.**
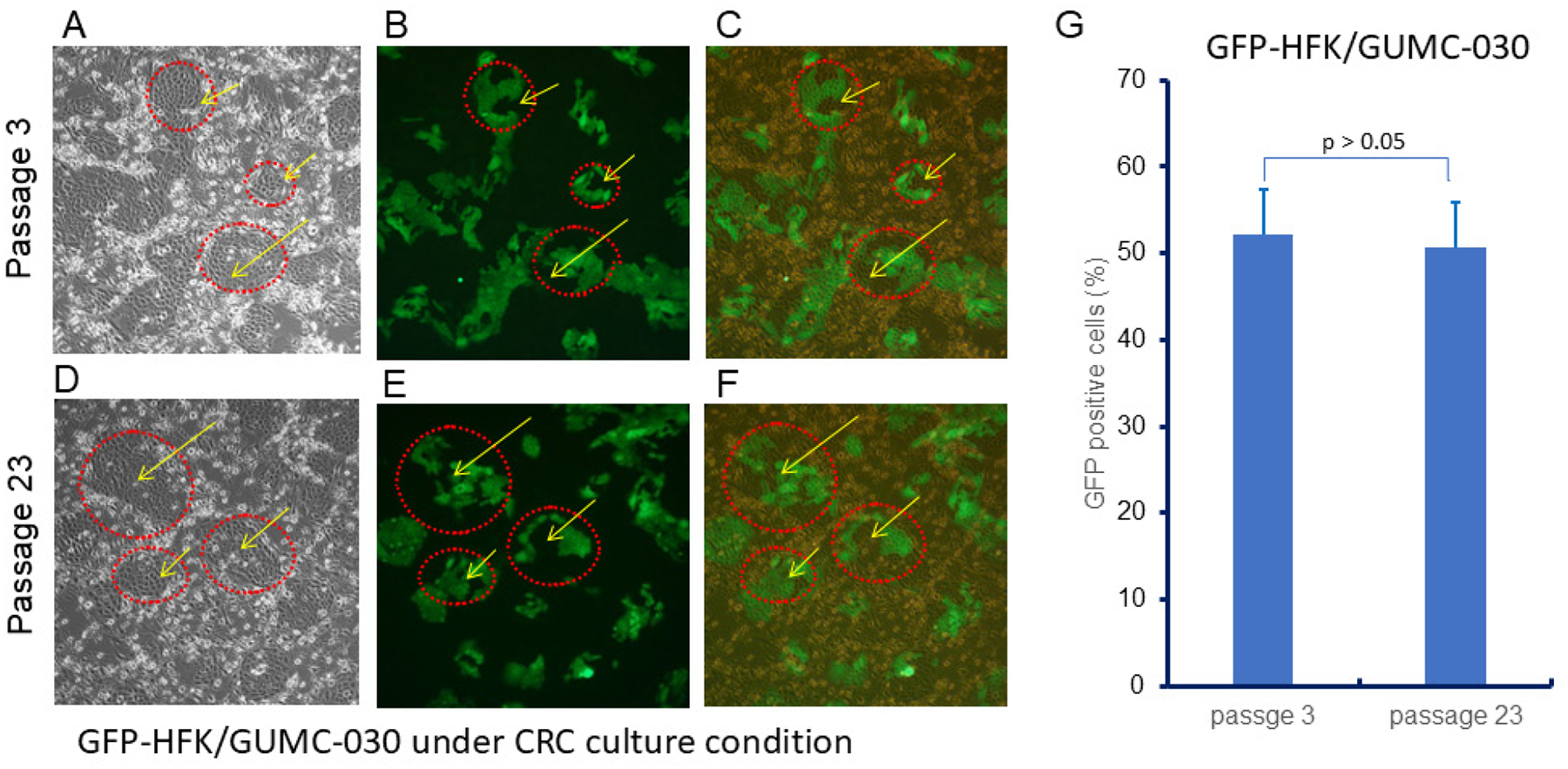
Co-culture of GFP-labeled HFKs and GUMC-030 cells in CRC conditions. Yellow arrows indicate mixed-cell colonies composed of both GFP-positive and GFP-negative cells. (A) Phase contrast microscopy of mixed GFP-HFKs and GUMC-030 at passage 3. (B) IF microscopy of mixed GFP-HFKs and GUMC-030 at passage 3. (C) Merged image of mixed GFP-HFKs and GUMC-030 at passage 3. (D) Phase contrast microscopy of mixed GFP-HFKs and GUMC-030 at passage 23. (E) IF microscopy of mixed GFP-HFKs and GUMC-030 at passage 23. (F) Merged image of mixed GFP-HFKs and GUMC-030 at passage 23. (G) Percentages of GFP-HFK (green) in mixed cultures at passages 3 and 23.

To evaluate the potential of CRCs to support mixed cultures, we co-cultured GFP-labeled HFKs with GUMC-030 cells at a 1:1 ratio. As shown in Figure 3, colonies formed in CRC cultures were composed of both GFP-positive (green) and GFP-negative cells, suggesting the formation of heterogeneous colonies with both GFP-HFK and GUMC-030 tumor cells (cycled and arrowed). We observed that these phenomena can be stably maintained from early and late passages (passage 3 and passage 23) as shown in Figure 3A, 3B, 3C vs 3D, 3E, 3F; and quantified using flow cytometry in Figure 3G. This model may be useful for studying tissue development and tumor heterogeneity in vitro.

### Optimizing Storage Conditions for Irradiated Feeder Cells

**Figure 4.**
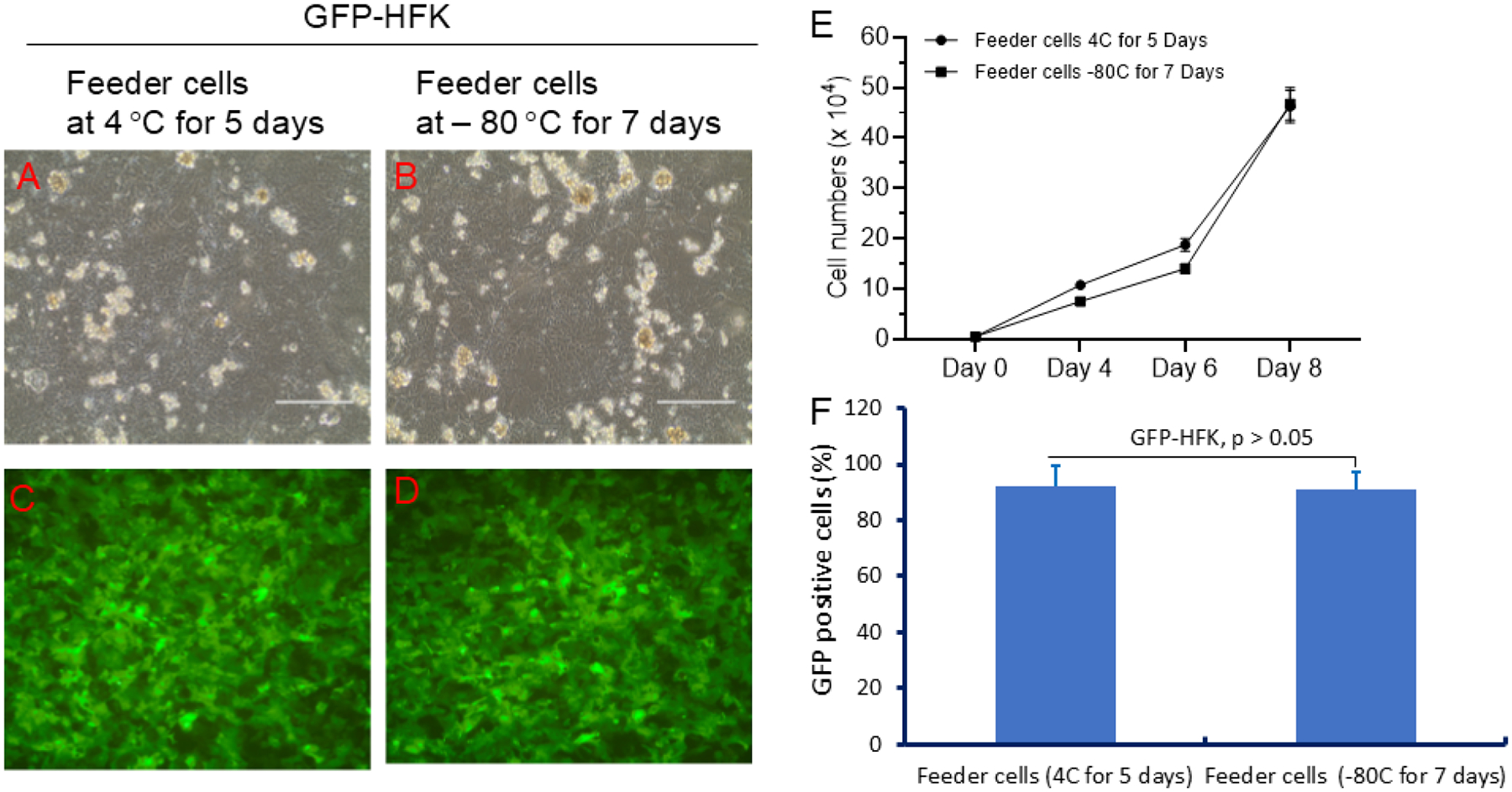
Irradiated feeder cell storage conditions for CRC. (A) Phase contrast microscopy of GFP-HFK with J2 cells stored at 4 °C for 5 days. (B). Phase contrast microscopy of GFP-HFK with J2 cells stored at −80 °C for 7 days. (C) IF microscopy of GFP-HFKs with J2 cells stored at 4 °C for 5 days. (D) IF microscopy of GFP-HFKs with J2 cells stored at −80 °C for 7 days. (E) Cell proliferation curve under two conditions. (F) Percentages of GFP-HFK in mixed GFP-HFK/GUMC-030 cultures using two sources of irradiated feeder cells. Both conditions supported GFP-HFK proliferation.

Since CRC culture requires irradiated Swiss 3T3 fibroblasts (J2) as feeder cells, we evaluated their stability after storage at 4 °C (5 days) and −80 °C (7 days). As shown in Figure 4, both storage conditions allowed the irradiated J2 cells to support GFP-HFK proliferation, as observed by fluorescence microscopy. This enables batch preparation of feeder cells for routine and long-term use.

### Human Fibroblasts as Animal-Free Alternatives in CRC

**Figure 5.**
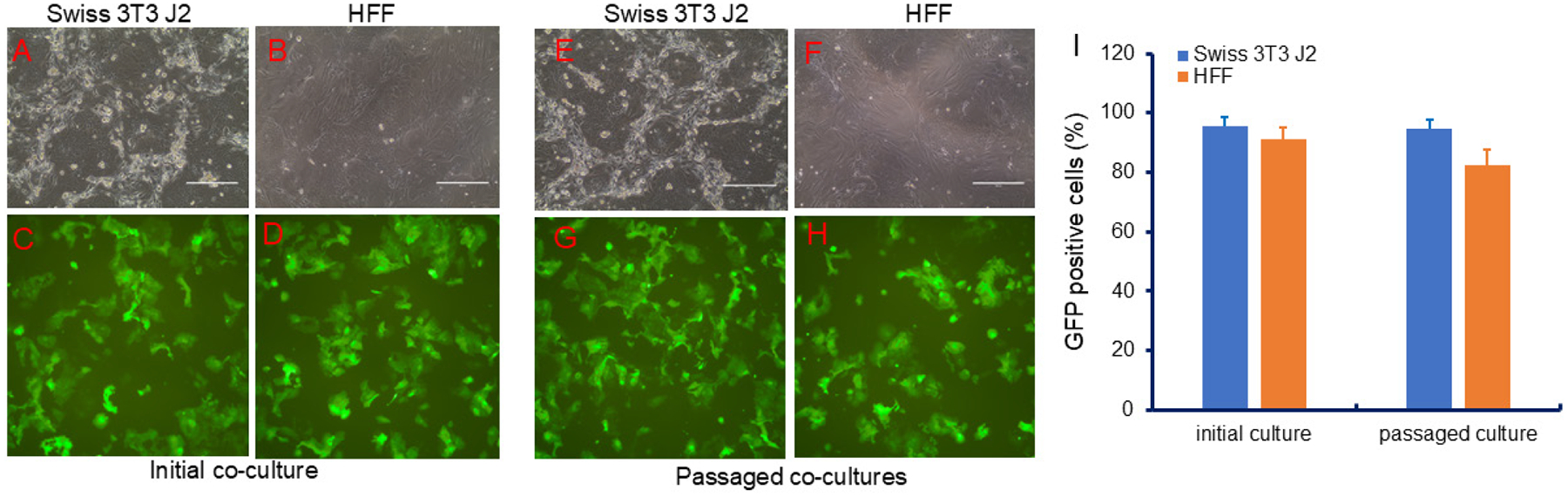
Human foreskin fibroblasts (HFFs) as feeder cells in GFP-HFK coculture. (A & E) Phase contrast microscopy of GFP-HFK with irradiated mouse Swiss 3T3 J2 cells. (B & F) Phase contrast microscopy of GFP-HFK with irradiated human foreskin fibroblasts (HFF). (C & G) Fluorescent microscopy of GFP-HFK with irradiated mouse Swiss 3T3 J2 cells. (D & H) Fluorescent microscopy of GFP-HFK with irradiated human foreskin fibroblasts (HFF). (I) Cell proliferation curves under two feeder cells (irradiated J2 feeders and Irradiated HFF feeders).

We tested irradiated human foreskin fibroblasts (HFFs) as feeder cells. As shown in Figure 5, HFFs supported the growth of GFP-HFKs, similar to J2 cells. Notably, HFFs remained attached after 5 days of co-culture, in contrast to J2 feeders, which began detaching after 2–3 days. We also noted that percentage of GFP positive cells slightly decreased as shown in Figure 5I measured with flow cytometry, and that borders of feeder cells surrounding HFK colonies were quite different from J2 and HFF (Figure 5A vs B, E vs F), while colonies in both feeder cells were not different as shown in both light images (Figure 5A vs 5B, 5E vs 5F) and fluorescent microimages (Figure 5C vs 5D, 5G vs 5H).

### Irradiation Dose Response of HFFs

**Figure 6.**
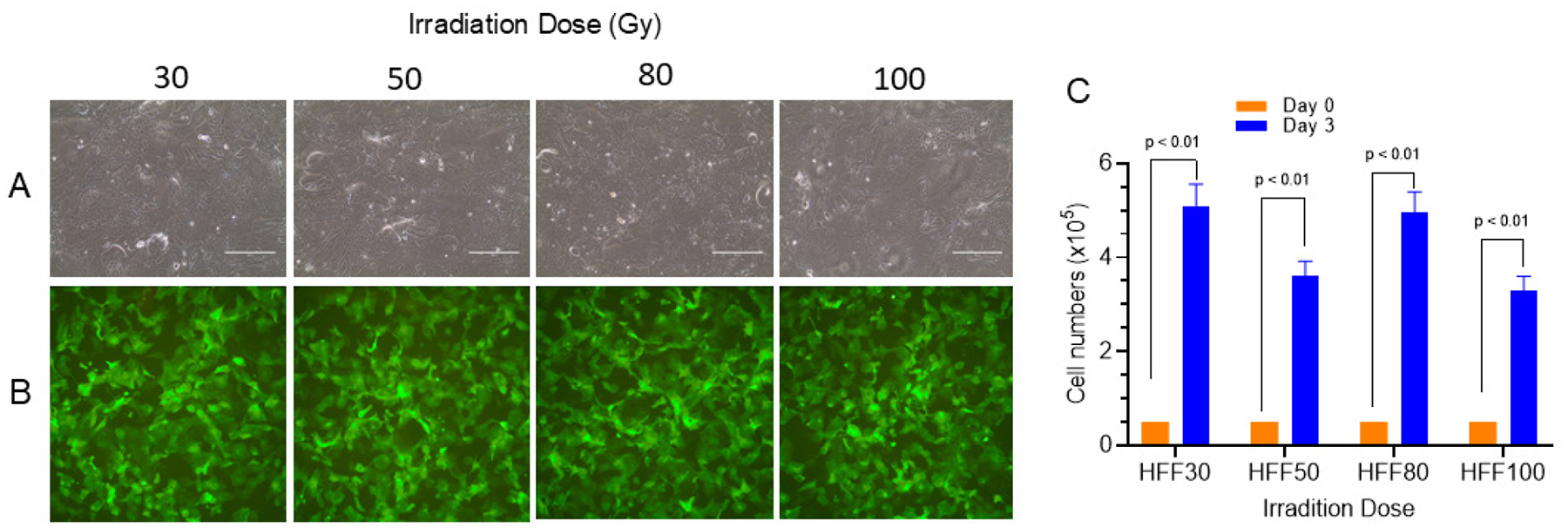
Irradiation dose of HFF feeders in CRC. (A) Phase contrast morphology of GFP-HFK with irradiated HFFs at different doses. (B) IF microscopy of GFP-HFK with irradiated HFFs at different doses. (C) GFP-HFK cell counts on Day 3 post-culture.

To determine whether irradiation dose affects feeder performance, HFFs were irradiated at doses ranging from 3,000 to 100,000 rad. As shown in Figure 6A, morphology remained consistent across doses. Figure 6B shows a similar density of GFP-positive cells under HFF cocultures. Quantitative cell counts (Figure 6C) showed no significant difference in GFP-HFK proliferation, suggesting that HFFs can be used as feeder cells across a wide irradiation range. This is very different from irradiation doses for J2 cells at 20–30 rad as we described previously.

### Tumor-Derived CRCs Can Be Genetically Manipulated

**Figure 7.**
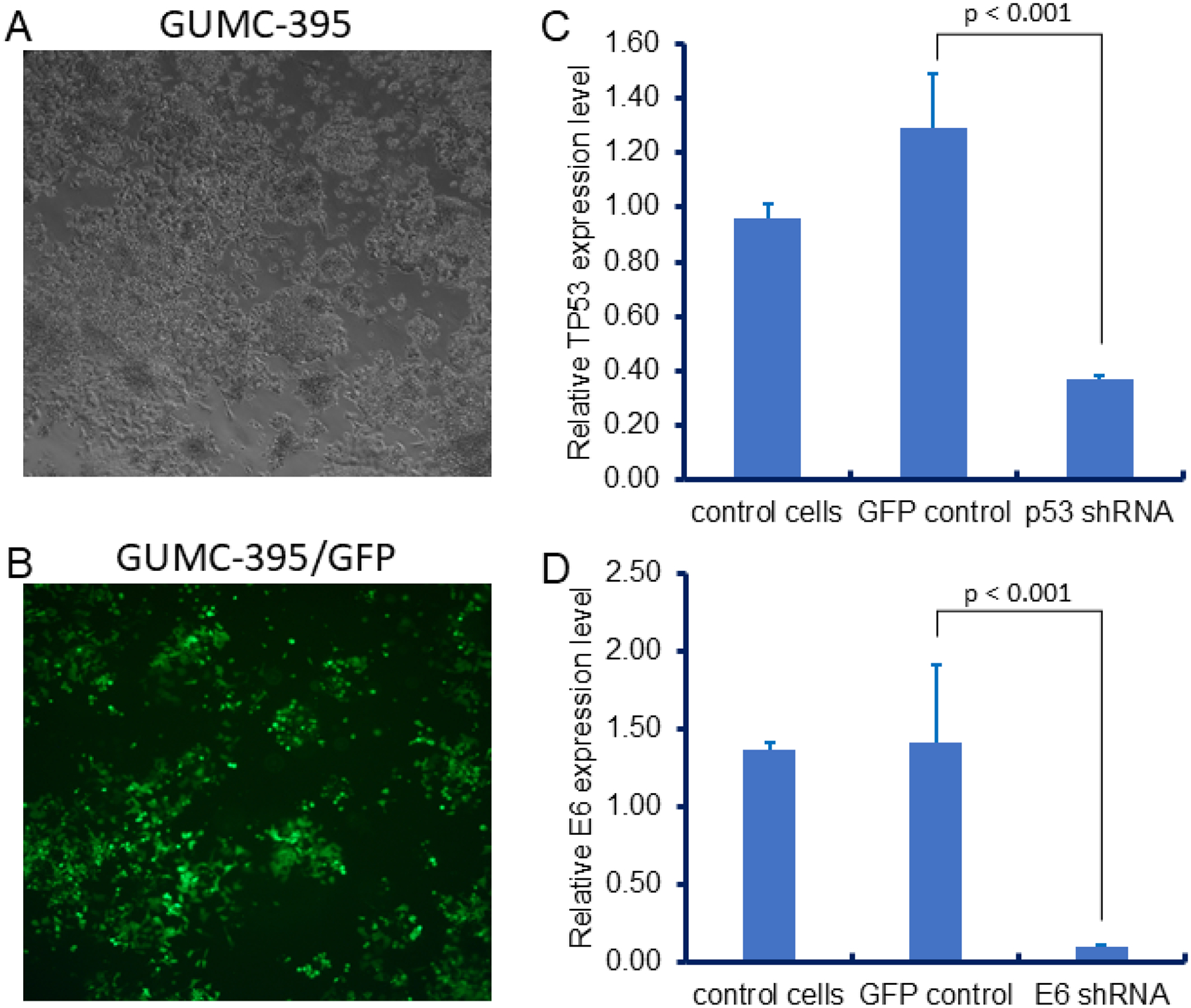
Genetic manipulation of tumor CRCs. (A) Phase contrast morphology of GUMC-395-GFP cells under CRC conditions. (B) IF morphology of GUMC-395-GFP cells under CRC conditions. RT-PCR validation of p53 (C) and E6 (D) knockdown following lentiviral shRNA infection.

We next assessed whether tumor CRCs can also be genetically manipulated. GUMC-395 cells were first transduced with GFP lentivirus to establish GUMC-395-GFP cells (Figure 7A and 7B). Subsequently, we infected these cells with shRNA lentiviruses targeting TP53 and HPV E6. RT-PCR confirmed successful knockdown of both genes (Figure 7C & 7D). These manipulations will allow further investigation into the roles of mutant p53 and HPV E6 in tumor proliferation. Together, these findings demonstrate that both normal and tumor CRCs are amenable to stable genetic modification. These data indicate that CRCs can be potentially used for cell therapies of genetic diseases, for example, cystic fibrosis, and cancer biology studies as well.

### Micro-heterogeneity of breast cancer using CRC and single-cell-derived clones.

Since our above results indicated the potential of CRC in tumor heterogeneity, we performed single-cell-derived CRC clones from a needle biopsy from a patient with breast cancer, as we described previously [20]. Needle biopsy tissue from a breast cancer patient was processed and digested as described previously. The resulting single-cell suspension was diluted to 1 cell per 200 μl, and 100 μl were plated per well across ten 96-well plates. All wells were evaluated under light microscopy; wells containing two or more cells were excluded from further analysis. Nine single-cell-derived clones were successfully expanded after sequential replating into 24-well and 12-well plates, and then into T25 flasks. DNA and RNA were isolated from five clones, along with adjacent normal cells and tumor CRCs, for whole-genome sequencing and transcriptome analyses [20].

### Clustering of differentially expressed genes between subgroups C1&C2 and C3&C6&C8.

**Figure 8.**
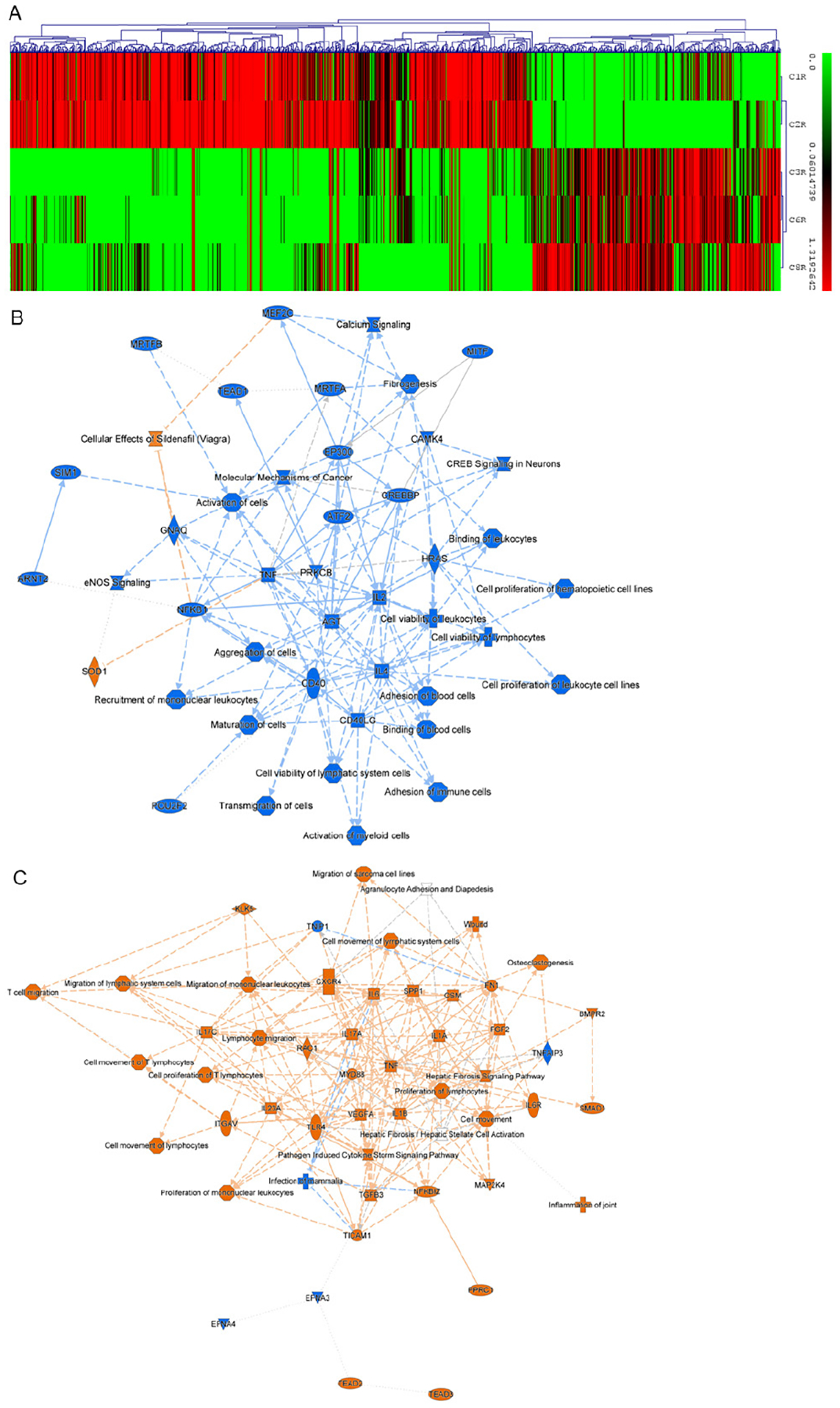
Micro-heterogeneity of breast cancer using CRC and single-cell-derived clones. (A) Heatmap of differentially expressed genes between subgroups C1&C2 and C3&C6&C8. The heatmap was constructed using MultiExperiment Viewer (MeV) v4.9.0. Both genes and samples were grouped via hierarchical clustering analysis (HCL) using Euclidean distance metrics. (B) Pathway network in subgroups C1 and C2. The network is dominated by pathways related to immune cell activation, survival, and proliferation, with central hubs including TNF, IL2, IL4, CD40/CD40LG, NFKB1, CREBBP, and EP300. Enriched functions include activation and viability of leukocytes and lymphocytes, calcium and CREB signaling, and transcriptional/epigenetic regulation, together with pathways associated with the molecular mechanisms of cancer. This network reflects a regulated, activation-driven immune environment closely linked to oncogenic and cell survival processes. (C) Pathway network in subgroup C3&C6&C8. This network is enriched for inflammatory, migratory, and tissue remodeling pathways, with prominent nodes such as TNF, IL1A, IL1B, IL6, IL17A, CXCR4, TLR4, MYD88, TGFB3, VEGFA, and FN1. Canonical pathways include pathogen-induced cytokine storm signaling, agranulocyte adhesion and diapedesis, lymphocyte and mononuclear leukocyte migration, hepatic fibrosis/stellate cell activation, and wound response. Collectively, this network represents a highly inflammatory, innate immune-dominated state associated with immune cell trafficking, stromal activation, and fibrotic remodeling. Together, these analyses highlight a shift from immune activation and survival signaling to inflammation-driven migration and tissue response.

Based on the subgroup classification shown above, we further selected differentially expressed genes that have different expression scales in subgroup C1 & C2 and subgroup C3 & C6 & C8. The number of selected genes is 1327 (Figure 8A). Genes exhibiting higher expression levels in C3R, C6R, and C8R were categorized as Cluster 1 (total genes: 428), whereas those with elevated expression in C1R and C2R were designated as Cluster 2 (total genes: 899). Both gene sets were subsequently subjected to Ingenuity Pathway Analysis (IPA). The further pathway analysis was shown in Figures 8B and 8C. The two pathway networks share inflammatory and immune-related signaling nodes but differ substantially in their biological emphasis, dominant pathways, and infer functional context. Figure 8B (C1 & C2) is centered on cell activation, survival, transcriptional regulation, and cancer-related signaling, with strong integration of immune cell viability and proliferation. The central hubs include TNF, IL2, IL4, CD40/CD40LG, NFKB1, CREBBP, EP300, ATF2, PRKCB, HRAS. This also indicates strong enrichment for activation of immune cells, cell viability, and proliferation (leukocytes, lymphocytes, hematopoietic cells), transcriptional and epigenetic regulation (CREB/CREBBP, EP300, ATF2), cancer-associated pathways, including Molecular Mechanisms of Cancer. Signaling pathways include NF-κB, CREB signaling, calcium signaling, and eNOS signaling. The functional themes include immune cell activation and survival, adaptive immune regulation, and oncogenic and transcriptional control networks. This network reflects a proliferative, activation-driven immune environment closely linked to tumor biology and cell survival, suggesting relevance to cancer initiation, immune modulation, and growth-supportive signaling. Figure 8C (C3, C6, and C8) is enriched for inflammatory, migratory, and tissue remodeling pathways, with prominent nodes such as TNF, IL1A, IL1B, IL6, IL17A, CXCR4, TLR4, MYD88, TGFB3, VEGFA, and FN1. Canonical pathways include pathogen-induced cytokine storm signaling, agranulocyte adhesion and diapedesis, lymphocyte and mononuclear leukocyte migration, hepatic fibrosis/stellate cell activation, and wound response. Collectively, this network represents a highly inflammatory, innate immune-dominated state associated with immune cell trafficking, stromal activation, and fibrotic remodeling. Together, these analyses highlight a shift from immune activation and survival signaling to inflammation-driven migration and tissue response. This contrast suggests a potential transition from immune activation to inflammatory remodeling, or distinct disease contexts (e.g., tumor-driven signaling versus inflammation-driven tissue response) in these two subpopulations.

These findings suggest that CR technology provides a powerful biological approach to studying the heterogeneity of breast cancer using patient-derived specimens, including limited samples from advanced breast cancer patients.

## Discussion

CR technology involves co-culturing epithelial cells with irradiated Swiss-3T3-J2 mouse fibroblasts (feeder cells) in the presence of the ROCK inhibitor Y-27632. Using this approach, primary keratinocytes, as well as prostate and mammary epithelial cells, can be reprogrammed into a basaloid, stem-like phenotype, forming well-organized prostaspheres and mammospheres in Matrigel [18, 26, 27]. The CR method rapidly induces both normal and tumor cells into a reprogrammed stem-like state, characterized by high proliferative capacity while retaining their original karyotypes. Importantly, once the CR conditions are removed, the cells’ ability to differentiate is restored, and the phenotype is fully reversible [26, 27]. CR has proven highly effective in generating large numbers of primary cells from diverse tissue sources, including fine-needle aspiration (FNA), core biopsies, surgical specimens, brushings, swabs, urine samples, and patient-derived xenograft tissues [26, 35, 44, 46]. Remarkably, CR technology allows the establishment and expansion of ~2 million epithelial cells in only 5–6 days, and cultures can be continuously passaged for up to 100 population doublings over ≥ 110 days [18, 47, 48].

CRCs have the capability to reflect the genetic and histological properties of the parental tissue, thereby enabling them to sustain a highly proliferative state [49]. An important aspect is the versatility of these cultures, which can be employed for establishing xenografts [27], patient-derived xenograft (PDX) cell lines [50], cell cultures derived from PDXs, and organoid cultures. Thus, CRC ensures the preservation of cell lineage commitment and the cellular heterogeneity inherent in a biopsy specimen [26, 51]. Modeling diseases, precision medicine, drug discovery, regenerative medicine, and noninvasive diagnosis are some of the applications of the CRC technique [24, 52, 53]. Clinical and translational research applications of CRC technology have been investigated in various diseases such as, lung cancer [40, 54–56], cystic fibrosis [37], upper respiratory viral infections [39], breast cancer [57], prostate cancer [58], bladder cancer [59], and digestive system disorders [60], laryngeal and hypopharyngeal cancers [61], nasopharyngeal carcinoma [62, 63], head and neck squamous carcinomas [64, 65]. Furthermore, CR technology has shown its potential in rare conditions such as respiratory papillomatosis [66] and salivary cancer [67]. Furthermore, the utility of this system extends beyond humans; it can be applied to various mammalian species, including mice, rats, ferrets, horses, cows, and dogs [59, 68].

In this study, we demonstrated efficient transduction in GUMC395 cells, a cervical large cell neuroendocrine carcinoma line, using GFP- and shRNA-expressing lentiviruses. shRNA knockdown of p53 and HPV16 E6 confirmed that genetic engineering does not compromise CR proliferation potential. Importantly, CRC conditions allow co-culture of distinct epithelial lineages (e.g., keratinocytes and glandular cells) or normal and tumor cells, without impairing growth or phenotype. This supports future applications in tissue regeneration and disease modeling.

Moreover, GUMC-395 was from a 26-year-old girl who was diagnosed with a rare large cell neuroendocrine carcinoma of the cervix. The histology of the primary suggested a very aggressive tumor (Ki67 staining 93%). Usually, in HPV positive cervical cancer, the E6-E6AP complex results in the ubiquitin-dependent degradation of p53; this is why we found loss of p53 protein [69]. However, we found two different p53 point mutations from two different portions of the tumor (the common R273C mutation). This background may give us a new perspective of new function of E6. Also, the invasive clinical behavior of a tumor may be explained by the combination of genetic abnormalities, e.g., Myc and IGH-BCL2 [70]. This HPV16 positive cervical tumor cells (GUMC395) also have 100 copies of the Myc gene amplification, which most likely contributes to the invasiveness. We can have answers for all these questions very soon using shRNAs against p53, HPV16 E6, and Myc as well.

Our data also indicated that normal HFKs expressing GFP can be co-cultured with prostate cancer without affecting each other’s growth and GFP expression. To address translational concerns with murine feeders, we tested human fibroblasts as alternatives. Both supported the proliferation of human keratinocytes [71, 72], reducing risks of pathogen transfer and immune rejection. These findings suggest CR could be adapted for clinical use, including regenerative therapies for diabetes, cardiovascular disease, and cancer [73–75].

Nakshatri et al. used CR to expand epithelial cells from > 60 normal breast tissues, identifying at least 20 cell subtypes per individual. CR supported the expansion of stem, progenitor, and mature cells, with notable differences between African American and Caucasian women. A unique CD44ĥigh/CD24^−/EpCAM^− population, enriched in African American samples, showed gene expression consistent with PROCR^+/EpCAM^− mammary stem cells [73, 74]. Our data (Figure 8) also demonstrated CRC as a valuable platform for studying tissue and tumor heterogeneity, especially from limited patient samples.

Traditional cell cultures typically rely on immortalized cell lines or primary cells with limited proliferative capacity that undergo senescence after a few passages. In contrast, CRC technology allows rapid and indefinite expansion of primary cells from patient tissues or fluids without genetic manipulation, maintaining diploid genomes and avoiding artificial immortalization. Conventional cell lines often exhibit altered differentiation states, aberrant signaling pathways, and reduced relevance to in vivo biology due to long-term adaptation to culture conditions. CRC cultures retain tissue-specific differentiation potential, lineage identity, and clinically relevant phenotypes, closely reflecting the original patient tissue. Traditional immortalization methods (e.g., viral oncogenes, hTERT) introduce genetic alterations that confound biological interpretation. CRC cultures preserve genomic integrity and epigenetic regulation, enabling more accurate studies of disease initiation, progression, and drug response. Finally, CRC cultures support personalized drug testing, biomarker discovery, and regenerative medicine applications, whereas traditional cultures are limited in translational relevance.

CRC technology bridges the gap between in vitro models and human disease, providing a physiologically relevant and patient-specific culture platform not achievable with conventional methods. There are several limitations: (1) Dependence on feeder cells and specialized culture conditions: since traditional CRC systems require irradiated feeder cells and ROCK inhibition, which increase technical complexity and may introduce variability. These components can complicate standardization and regulatory translation, particularly for clinical-grade applications. (2) Incomplete recapitulation of the tumor microenvironment: CRC cultures primarily expand epithelial cells and lack stromal, immune, and vascular components, limiting their ability to fully model cell-cell interactions, immune modulation, and microenvironment-driven therapeutic responses. (3) Selection bias during cell expansion: CRC conditions may preferentially expand certain epithelial subpopulations, potentially underrepresenting rare clones or highly differentiated cells present in the original tissue, which may affect studies of intratumoral heterogeneity. (4) Loss of in vivo architecture and spatial context: as a two-dimensional culture system, CRCs do not preserve native tissue architecture or polarity, which can influence differentiation, signaling pathways, and drug responses. (5) Challenges in tumor-normal discrimination: In samples containing mixed cell populations (e.g., urine, biopsy specimens), CRC conditions can support robust growth of both normal and tumor epithelial cells, requiring additional molecular characterization to distinguish malignant from non-malignant cultures. 6. Inter-laboratory variability and standardization. Differences in feeder preparation, media composition, and handling can lead to variability across laboratories, posing challenges for multi-center studies and large-scale clinical deployment.

In conclusion, while CRC technology offers unparalleled advantages in expanding patient-derived epithelial cells with high biological fidelity, these limitations highlight the need for standardization, feeder-free systems, integration with 3D or co-culture models, and rigorous molecular validation to support broader clinical translation.
